# Impact of Time-of-Flight and Point-Spread-Function for Respiratory Artifact Reduction in PET/CT Imaging: Focus on Standardized Uptake Value

**Published:** 2017

**Authors:** Roya Sharifpour, Pardis Ghafarian, Mehrdad Bakhshayesh-Karam, Hamidreza Jamaati, Mohammad Reza Ay

**Affiliations:** 1 Department of Medical Physics and Biomedical Engineering, Tehran University of Medical Sciences, Tehran, Iran,; 2 Research Center for Molecular and Cellular Imaging, Tehran University of Medical Sciences, Tehran, Iran,; 3 Chronic Respiratory Diseases Research Center, National Research Institute of Tuberculosis and Lung Diseases (NRITLD), Shahid Beheshti University of Medical Sciences, Tehran, Iran,; 4 PET/CT and Cyclotron Center, Masih Daneshvari Hospital, Shahid Beheshti University of Medical Sciences, Tehran, Iran,; 5 Pediatric Respiratory Diseases Research Center, National Research Institute of Tuberculosis and Lung Diseases (NRITLD), Shahid Beheshti University of Medical Sciences, Tehran, Iran.

**Keywords:** Standardized Uptake Value, Threshold, Time-of-Flight, Point-Spread-Function, PET/CT imaging, Respiratory artifact

## Abstract

**Background:**

The most important advantage of positron emission tomography/computed tomography (PET/CT) imaging is its capability of quantitative analysis. The aim of the current study was to choose the proper standardized uptake value (SUV) threshold, when the time-of-flight (TOF) and point spread function (PSF) were used for respiratory artifact reduction in the liver dome in a new-generation PET/CT scanner.

**Materials and Methods:**

The current study was conducted using a National Electrical Manufacturers Association International Electrotechnical Commission body phantom, with activity ratios of 2:1 and 4:1. A total of 27 patients, with respiratory artifacts in the thorax region, were analyzed. PET images were retrospectively reconstructed using either a high definition (HD) + PSF (i.e., a routine protocol) algorithm or HD+PSF+TOF (PSF+TOF; i.e., to reduce the respiratory artifact) algorithms, with various reconstruction parameters. The SUV_max_ and SUV_mean_, at different thresholds (i.e., at 45%, 50%, and 75%), were also assessed.

**Results:**

Although in comparison to the routine protocol a higher SUV was observed when using the PSF+TOF method, this approach was used to reduce the respiratory artifact. The appropriate threshold for SUV was strongly related to the lesion size, reconstruction parameters, and activity ratio. The mean of the relative difference between PSF+TOF algorithm and routine protocol for SUV_max_ varied from 10.58±14.99% up to 35.49±32.60% (which was dependent on reconstruction parameters).

**Conclusion:**

In comparison with other types of SUVs, the SUV_max_ value illustrated its significant overestimation, especially at the 4:1 activity ratio. The poor agreement between SUV_max_ and SUV_50%_ was also observed. When the TOF and PSF are utilized to reduce respiratory artifacts, the SUV_50%_ can be an accurate semi-quantitative parameter for PET/CT images, for all lesion sizes. For smaller lesions, however, a smaller filter size was required to observe an accurate SUV.

## INTRODUCTION

The quantitative assessment of ^18^F-fluorodeoxyglucose (^18^F-FDG) positron emission tomography/computed tomography (PET/CT) imaging is a noninvasive tool with applications in oncology. Thus, metabolic and volumetric parameters lead to valuable data for patient monitoring and evaluation of therapy response (1–3). The standardized uptake value (SUV) is a semi-quantitative parameter, which is widely used in evaluation of glucose metabolism, is prone to biological and technical factors (4–6). The SUVs, however, can vary depending on the method of reconstruction and parameters that are used (7–11). Thus, since PET images are obtained from different scanners, or even with different reconstruction methods in the same scanner, an accurate SUV is essential for reliable quantitative analysis. Previous studies have suggested different approaches to address reconstruction-dependent variation in SUVs (12–14). Furthermore, a more accurate SUV can be obtained with better spatial resolution (15, 16). The point spread function (PSF) has been used during PET reconstruction to improve the spatial resolution (17). Moreover, the time-of-flight (TOF) and PSF functions in new-generation PET/CT scanners lead to better lesion detectability, signal to noise ratio (SNR), spatial resolution, and uniformity (15, 16, 18–22). However, these techniques can also lead to higher SUVs (10, 11, 19, 23, 24). Isocontour thresholds, the sizes and shapes of the volume of interest (VOI), and various correction methods can provide different SUVs for the same lesion (7, 25–29). Previous studies have demonstrated that errors in the SUV measurement can lead to variations of up to 50% (4, 25, 26). In our previous study (30), we showed that, in the absence of gating devices, the TOF technique can reduce the respiratory artifact in the liver dome. In the current study, we aimed to extend our results by evaluating the combination of TOF and PSF with various reconstruction parameters to choose the optimal SUV threshold for clinical PET/CT images, for accurate quantification. This evaluation was performed to quantify PET/CT images with indices consisting of SUV_max_ and SUV_mean_ with various thresholds, in systematic phantom and patient studies.

## MATERIALS AND METHODS

### PET/CT Scanner

All measurements were performed on a Discovery 690 VCT (GE Healthcare, Milwaukee, USA) scanner, which was equipped with 64-slice CT (Light Speed VCT). The 58368 solid state detector elements of the CT scanner were arranged in 912 channels, in 64 rows. The PET component of the Discovery 690 VCT used 4.2×6.3×25 mm^3^ lutetiumyttrium oxyorthosilicate (LYSO) crystals. The PET scanner consisted of 24 detector rings, with a 157 mm axial field of view (FOV). In the PET scanner, the timing resolution and coincidence time window of the TOF method were approximately 500 ps and 4.9 ns, respectively.

### Phantom Study

A National Electrical Manufacturers Association (NEMA) International Electrotechnical Commission (IEC) Body Phantom was used in the current study. This phantom contained six spherical inserts (with internal diameters of 10, 13, 17, 22, 28, and 37 mm) to simulate tumors with different sizes, and one cylindrical insert to simulate the lung. The background of phantom was filled with 5.3 KBq/mL ^18^F-FDG solution. All spheres were filled with activity ratios of 4:1 and 2:1, relative to the background activity separately.

### Patients Population

We retrospectively assessed 27 patients (i.e., 16 men and 11 women). The patients were the same as patient group in our previous study (30). The clinical indications for PET/CT examinations were as follows: lung cancer (n=4), non-Hodgkin’s lymphoma (n=4), Hodgkin’s lymphoma (n=3), colon cancer (n=2), renal cell carcinoma (n=4), pancreatic cancer (n=1), gastric cancer (n=1), breast cancer (n=3), esophageal cancer (n=4), and unknown primary cancer (n=1). A total of 75 lesions, located in the diaphragmatic region, including the lower lobe of the lungs, liver, spleen, and stomach, were assessed. The patients had an average age of 55±15.21 years (range: 28–71 years). Patients with fasting blood sugar levels higher than 200 mg/dL were excluded from the current study. The fasting period was between 6 to 8 h. The PET/CT scan was performed 60.75±1.48 min after an intravenous injection of 331.42±71.03 MBq (range: 253–487 MBq) of ^18^F-FDG, according to the European Association of Nuclear Medicine guidelines (31).

### Data Acquisition and Image Reconstruction

Following CT acquisition using smart mA technique, all emission data were obtained from the vertex to mid-thigh. PET images were reconstructed using a 256×256 image matrix, with 2.73 mm pixels, for the two main groups consisting of the routine protocol (with respiratory artifacts in the liver dome) and PSF+TOF (to reduce the artifacts). The VUE point high definition (HD) with PSF, (HD+PSF), was used as our routine protocol. The VUE point FX with PSF, (HD+PSF+TOF), was used as the PSF+TOF method. The reconstruction parameters used for our routine protocol were 3 iterations and 18 subsets, with a 6.4 mm Gaussian filter in full width at half maximum (FWHM). The PSF+TOF methods were reconstructed with 2 iterations, and 18 and 24 subsets, with 4.4, 5.4, and 6.4 mm FWHM post-smoothing filters.

### Assessment Strategy

The current study aimed to obtain accurate quantification. For this purpose, the SUV_max_ (3D isocontour encompass the total lesion), and SUV_45%_, SUV_50%_, and SUV_75%_ (with the mean 3D isocontour values at 45%, 50%, and 75% of the maximum voxel value, respectively) were measured in the PET images. This was repeated for all reconstruction methods for both phantom and clinical data. The relative difference for all types of SUV and mean of difference in the SUV value for various reconstruction methods and routine protocol were also calculated.

### Statistical Analysis

Statistical analyses were performed using SPSS packages (SPSS, version 22.0, Armonk, NY). The Shapiro-Wilk method was utilized to test for normality. A paired t-test was used for variables with a normal distribution, while a Wilcoxon signed ranks test was conducted on non-normally distributed data. The Lin concordance correlation coefficient (Lin CCC) was also applied to test the agreement between variables. Statistical significance was set at p<0.05.

## RESULTS

**Figure 1.**
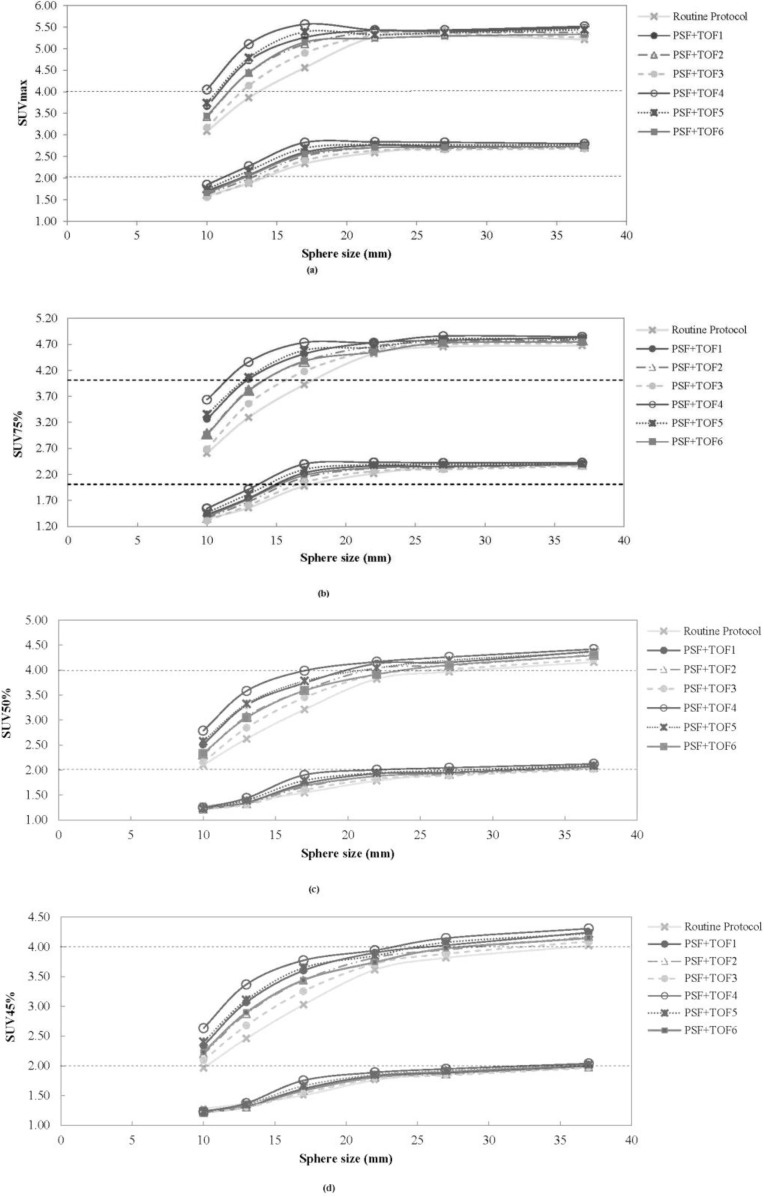
Illustration of measured (a) SUVmax, (b) SUV75%, (c) SUV50% and (d) SUV45% as a function of various sphere sizes in NEMA IEC Body phantom with 4:1 (upper) and 2:1(lower)activity ratio and background concentration of 5.31kBq/mL. Routine Protocol (HD+PSF with 3it, 18sub,6.4mm filter), PSF+TOF1(2it, 18sub, 4.4mm filter), PSF+TOF2(2it, 18sub, 5.4mm filter), PSF+TOF3(2it, 18sub, 6.4mm filter), PSF+TOF4(2it, 24sub, 4.4mm filter), PSF+TOF5(2it, 24sub, 5.4mm filter), PSF+TOF6(2it, 24sub, 6.4mm filter).

**Figure 2.**
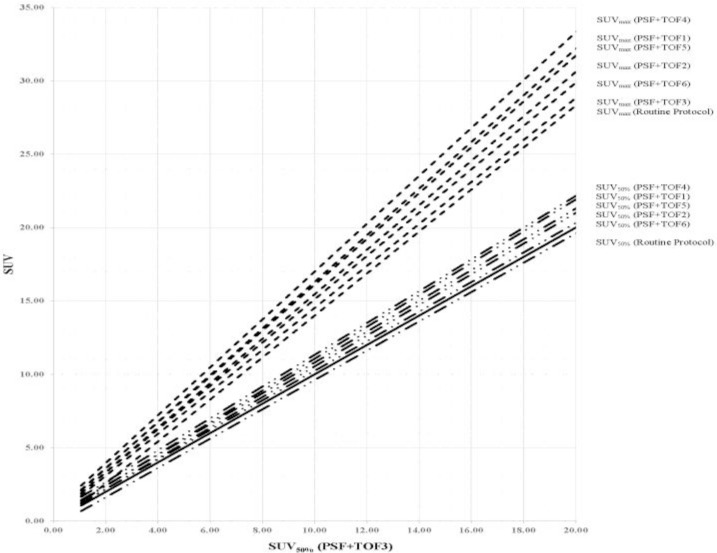
Correlation between measured SUV of 27 patients data. SUV50% with regard to PSF + TOF3 method is compared with the other reconstruction methods. The solid line shows the identity line. Routine Protocol (HD+PSF with 3it, 18sub,6.4mm filter), PSF+TOF1(2it, 18sub, 4.4mm filter), PSF+TOF2(2it, 18sub, 5.4mm filter), PSF+TOF3(2it, 18sub, 6.4mm filter), PSF+TOF4(2it, 24sub, 4.4mm filter), PSF+TOF5(2it, 24sub, 5.4mm filter), PSF+TOF6(2it, 24sub, 6.4mm filter).

**Figure 3.**
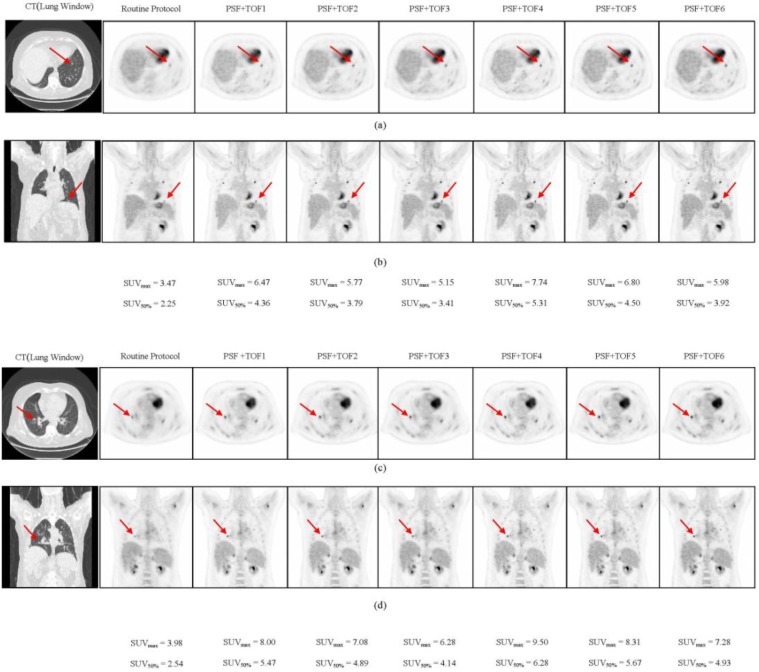
Top (a,b) and bottom (c,d) pulmonary nodulesof a patient with colon cancer.Transverseandcoronal view forlung window of CT images and PET images with various reconstruction methods are illustrated. The impact of TOF on improvement in contrast and lesion detectability is clear in all methods. (Body weight: 105 kg and injected dose: 492MBq of ^18^F-FDG)

Figure 1 illustrates the comparison of the SUV_max_, SUV_75%_, SUV_50%_, and SUV_45%_ of each sphere size, between various reconstruction protocols in the phantom study. Decreasing the VOI threshold, extended the underestimation of all types of SUV from the small sphere sizes to the larger sphere size. Thus, the underestimation of SUV_45%_ for all sphere sizes was observed in the NEMA phantom with the 2:1 activity ratio. Compared with other SUV types, the SUV_max_ was significantly overestimated during the 4:1 activity ratio. As in our previous work (30), the PSF+TOF3 (2 iterations, 18 subsets, and 6.4-mm post-filter) showed the lowest noise level and highest SNR among of all reconstruction methods used in the current study. Figure 2 illustrates the correlation between SUV_50%_ and SUV_max_ for all clinical data between PSF+TOF3 (2 iterations, 18 subsets, and 6.4-mm post-filter) and the other reconstruction methods. A noticeable variation was observed between the SUV_max_ and SUV_50%_ for all reconstruction parameters. The Lin CCC that was used is listed in Table 1. Prieto et al. (11) stated that, although the correlation could be a necessity, it may not be enough to ensure agreement of values for quantitative purposes. Thus, poor agreement between the SUV_max_ and SUV_50%_ (Lin CCC ≤ 0.90) of various reconstruction methods was seen. However, moderate, substantial, and almost perfect agreement was also observed (Lin CCC ≥ 0.90) between SUV_50%_ and SUV_max_ separately for each reconstruction method. In our clinical study, the mean of difference for various SUV thresholds between all reconstruction parameters and routine protocol is illustrated in Table 2. Decreasing the threshold diminished the differences, with a greater impact on small subset numbers and the larger post-smoothing filter. Figure 3 illustrates the lung window of CT images and PET images, with various reconstruction methods, of a typical patient with colon cancer. The reduction in the respiratory artifact was obvious when the PSF+TOF methods were applied. Furthermore, these methods led to a larger SUV_max_, compared with the SUV_50%_ for all corresponding reconstruction methods. In comparison with the routine protocol, the hypermetabolic pulmonary nodules were more varied for the SUV_max_ versus SUV_50%_ for the other reconstruction methods.

**Table 1.** Lin concordance correlation coefficients Between the SUV Measured with Different Protocols

		**SUV_max_**							**SUV_50%_**						

		**Routine Protocol**	**PSF+TOF1**	**PSF+TOF2**	**PSF+TOF3**	**PSF+TOF4**	**PSF+TOF5**	**PSF+TOF6**	**Routine Protocol**	**PSF+TOF1**	**PSF+TOF2**	**PSF+TOF3**	**PSF+TOF4**	**PSF+TOF5**	**PSF+TOF6**
**SUV_max_**	**Routine Protocol**	**---**	**---**	---	---	**---**	---	---	---	---	---	---	---	---	---
	**PSF+TOF1**	**0.95**	**---**	---	---	**---**	---	---	---	---	---	---	---	---	---
	**PSF+TOF2**	**0.97**	**0.99**	---	---	**---**	---	---	---	---	---	---	---	---	---
	**PSF+TOF3**	**0.99**	**0.98**	**0.99**	---	**---**	---	---	---	---	---	---	---	---	---
	**PSF+TOF4**	0.91	**0.99**	**0.98**	**0.95**	**---**	---	---	---	---	---	---	---	---	---
	**PSF+TOF5**	**0.95**	**1.00**	**0.99**	**0.98**	**0.99**	---	---	---	---	---	---	---	---	---
	**PSF+TOF6**	**0.97**	**0.99**	**1.00**	**1.00**	**0.97**	**0.99**	---	---	---	---	---	---	---	---
**SUV_50%_**	**Routine Protocol**	0.84	0.70	0.74	0.79	0.65	0.70	0.75	---	---	---	---	---	---	---
	**PSF+TOF1**	0.92	0.82	0.86	0.90	0.77	0.82	0.87	**0.95**	---	---	---	---	---	---
	**PSF+TOF2**	0.89	0.77	0.82	0.86	0.73	0.78	0.83	**0.97**	**0.99**	---	---	**---**	---	---
	**PSF+TOF3**	0.86	0.73	0.78	0.83	0.68	0.73	0.79	**0.99**	**0.98**	**0.99**	---	**---**	---	---
	**PSF+TOF4**	0.92	0.84	0.87	0.91	0.80	0.84	0.89	0.92	**0.99**	**0.98**	**0.96**	**---**	---	---
	**PSF+TOF5**	0.90	0.79	0.84	0.88	0.75	0.80	0.85	**0.95**	**1.00**	**1.00**	**0.98**	**0.99**	---	---
	**PSF+TOF6**	0.87	0.75	0.80	0.84	0.71	0.76	0.81	**0.98**	**0.99**	**1.00**	**1.00**	**0.98**	**0.99**	---

Lin Concordance Correlation Coefficients (CCC) greater than 0.95 are in bold.

**HD+PSF with 3it, 18sub and 6.4 mm filter**

**2it, 18sub, 4.4 mm filter**

**2it, 18sub, 5.4 mm filter**

**2it, 18sub, 6.4 mm filter**

**2it, 24sub, 4.4 mm filter**

**2it, 24sub, 5.4 mm filter**

**2it, 24sub, 6.4 mm filter**

**Table 2.** The Mean of Difference Between the SUV Measured with Various Protocols and Routine Protocol

**SUV**	**SUVmax**	**SUV75%**	**SUV50%**	**SUV45%**
**Various Protocols**				
PSF+TOF1^a^	1.47±1.06	1.31±0.97	1.01±0.73	0.93±0.67
PSF+TOF2^b^	1.00±0.77	0.87±0.66	0.68±0.54	0.64±0.48
PSF+TOF3^c^	0.52±0.57	0.45±0.49	0.38±0.39	0.36±0.35
PSF+TOF4^d^	1.95±1.43	1.74±1.33	1.28±0.93	1.21±0.89
PSF+TOF5^e^	1.41±1.09	1.23±0.92	0.93±0.73	0.88±0.69
PSF+TOF6^f^	0.88±0.89	0.73±0.68	0.58±0.56	0.55±0.51

a. **2it, 18 sub, 4.4 mm filter.**

b. **2it, 18 sub, 5.4 mm filter.**

c. **2it, 18 sub, 6.4 mm filter.**

d. **2it, 24 sub, 4.4 mm filter.**

e. **2it, 24 sub, 5.4 mm filter.**

f. **2it, 24 sub, 6.4 mm filter.**

## DISCUSSION

In the current study, we utilized a NEMA IEC body phantom to obtain the optimal SUV threshold for various sphere sizes and activity ratios, with various reconstruction methods (i.e., routine protocol and PSF+TOF). In the next step, the ability of various SUV thresholds to reduce the respiratory artifact was assessed in patients with different-sized lesions in the thorax region. Previous studies reported that using the PSF and TOF can lead to accurate quantitative analysis, with some SUV overestimation (10, 11). Thus, we obtained some optional reconstruction parameters to reach the acceptable image quality in oncology patients undergoing PET/CT imaging (21, 22). Variations in the reconstruction parameters and definition of the VOI for the SUV calculation can lead to discrepancies in the PET images properties and quantification analysis (28, 32). In the current study, we used SUV_max_, SUV_75%_, SUV_50%_, and SUV_45%_, for the quantitative analysis of PET images. When a specific SUV threshold was applied, the use of different reconstruction methods did not significantly change the 2:1 and 4:1 activity ratios in the NEMA phantom, of the 22-, 28-, and 37-mm sized spheres. It should be noted, however, that when the VOI threshold decreased, the SUV underestimation was extended to a bigger sphere size in both activity ratios. For the 2:1 and 4:1 activity ratios, the underestimation of the SUV_max_ and SUV_75%_ occurred for sphere sizes up to 10 mm and 17 mm, respectively, while those for SUV_50%_ and SUV_45%_ occurred for spheres sized 22 and 17 mm, and 28 and 22 mm, respectively. The results of our phantom pertaining to the overestimation of the SUV_max_ corroborated with the findings of Prieto et al. (11), Boellaard (33), and Kelly and Declerck (12). The SUV_max_ is a popular quantification parameter, which is used in 91% of diagnostic reports (34). The overestimation of this quantitative parameter can be attributed to the high noise level in small voxel sizes and the Gibbs effect (slight enhancement of the edges due to PSF modeling) (11). Our results demonstrated that the SUV threshold was dependent on both the activity ratio and lesion size. The least variation in SUV, compared with the routine protocol, was observed for PSF+TOF3, while the second and third level belonged to PSF+TOF2 and PSF+TOF6 in the 4:1 activity ratio. Similar behavior was seen for all reconstruction methods for the 2:1 activity ratio. According to our phantom study, among all SUV threshold, the SUV_50%_ could be an accurate quantitative parameters for spheres sized 22, 28, and 37 mm, for both 2:1 and 4:1 activity ratios, regardless of the reconstruction methods. A SUV_50%_ with a smaller filter size and higher subset number is more appropriate for smaller sphere sizes for the 4:1 activity ratio. In general, all reconstruction parameters illustrated nearly the same behavior for all sphere sizes, when the SUV_50%_ was used for the 2:1 activity ratio. Our clinical data corroborated with our phantom study; decreasing the filter size and increasing the subset number produced a high SUV for all thresholds in various lesions (PSF+TOF4 illustrated the highest SUV). The current study demonstrated that the SUV_max_ and SUV with different thresholds were strongly dependent on reconstruction parameters that in line with previous study (35). This effect, however, was less pronounced when a larger filter size and smaller subset number were used. The mean of relative difference (data were not shown) between PSF+TOF algorithm and routine protocol for SUV_max_ varied from 10.58±14.99% (PSF+TOF3; 2 iterations, 18 subsets, 6.4 mm post-filter) up to 35.49±32.60% (PSF+TOF4; 2 iterations, 24 subsets, 4.4 mm post filter). It should be noted that although accurate SUVs were observed for small lesions when smaller filter sizes were used, it did not seem critical for larger lesion sizes. However, the SUV_50%_ displayed variation when different reconstruction methods were used; it is an accurate semi-quantitative parameter for PET/CT images when the TOF and PSF were used to reduce the respiratory artifact for all lesion sizes. A few limitations of our study should also be noted. Since we did not have a phantom that simulated the respiratory cycle, we utilized a stationary NEMA phantom. Furthermore, since there were no heavy and obese patients, the effect of various reconstruction parameters could not be evaluated in those groups of patients.

## CONCLUSION

The PSF+TOF approach, with various reconstruction parameters produced higher SUVs (when compared with the routine protocol) and reduced the respiratory artifact in the liver dome. The accurate estimation of the SUV was strongly dependent on reconstruction parameters, lesion size, and activity ratios. There was less variation in the SUV between PSF+TOF algorithms and routine protocol when a smaller subset number and larger filter size were used. Regardless of the reconstruction method, compared with other SUV types, the SUV_50%_ illustrated more accurate quantitative parameters for PET/CT images when the TOF and PSF were applied to reduce the respiratory artifact for larger lesion sizes. A smaller filter size needs to be properly applied for smaller lesions with a high activity ratio.

## Abbreviations

Time of Flight=: TOF;
Point spread function=: PSF;
Positron emission tomography/Computed tomography=: PET/CT;
Standardized uptake value=: SUV;
^18^F-fluorodeoxyglucose=: ^18^F-FDG;
Signal to noise ratio=: SNR;
Volume of interest=: VOI;
Lutetium-yttrium oxyorthosilicate=: LYSO;
Field of view=: FOV;
National Electrical Manufacturers Association=: NEMA;
International Electrotechnical Commission=: IEC;
European Association of Nuclear Medicine=: EANM;
High definition =: HD;
Full width at half maximum=: FWHM;
Lin concordance correlation coefficient=: Lin CCC;
